# RelB plays an oncogenic role and conveys chemo-resistance to DLD-1 colon cancer cells

**DOI:** 10.1186/s12935-018-0677-x

**Published:** 2018-11-13

**Authors:** Xiaojun Zhou, Zhili Shan, Hengying Yang, Jingjing Xu, Wenjing Li, Feng Guo

**Affiliations:** 1 Department of General Surgery, The First Affiliated Hospital of Soochow University, Suzhou, 215006 China; 2 Center for Clinical Laboratory, The First Affiliated Hospital of Soochow University, Suzhou, 215006 China; 3 Department of Clinical Laboratory, Nanjing Medical University Affiliated Suzhou Hospital, Suzhou, 215006 China; 4 Department of Oncology, Nanjing Medical University Affiliated Suzhou Hospital, Baita West Road 16, Suzhou, 215001 China

**Keywords:** RelB, Colorectal cancer, Migration and invasion, Chemo-sensitivity, Cell cycle, Prognostic factor

## Abstract

**Background:**

Nuclear transcription factor kappa B (NF-κB) subunits exhibit crucial roles in tumorigenesis and chemo-sensitivity. Recent studies suggest that RelB, the key subunit of the alternative NF-κB pathway, plays a critical role in the progression of diverse human malignancies. However, the significance of RelB in colorectal cancer (CRC) remains unclear. Here, we systematically explored the functions of the alternative NF-κB subunit RelB in colon cancer cells and its underlying mechanism.

**Methods:**

Stably transfected RelB-shRNA DLD-1 cells were established using Lipofectamine 2000. NF-κB DNA-binding capability was quantified using an ELISA-based NF-κB activity assay. Cell growth was monitored by an x-Celligence system. Cell proliferation was analyzed by a CCK-8 and a Brdu proliferation assay. Response to 5-FU was assessed by an x-Celligence system. Cell apoptosis and cell cycle was detected using flow cytometry analyses. Cell migration and invasion abilities were detected by an x-Celligence system, Transwell inserts, and wound-healing assays. RelB expression and its clinical significance were analyzed using the CRC tissue microarray. The expression of NF-κB signaling subunits, AKT/mTOR signaling molecules, cell cycle related proteins, MMP2, MMP9, and Integrin β-1 were measured by Western blotting analyses.

**Results:**

The RelB-silencing inhibited cell growth of DLD-1 cells. The RelB-silencing exerted the anti-proliferative by downregulation of AKT/mTOR signaling. The RelB-silencing caused G_0_–G_1_ cell cycle arrested likely due to decreasing the expression of Cyclin D1 and CDK4, concomitant with increased expression of p27^Kip1^. The RelB-silencing enhanced cytotoxic effect of 5-FU and induced cell accumulation in S-phase. The RelB-silencing impaired the migration and invasion potential of DLD-1 cells, which was related to downregulation of MMP2, MMP9, and Integrin β-1. Importantly, the RelB expression was correlated with depth of tumor invasion, lymph node metastasis, metastasis stage, and pTNM stage. High-RelB expression was significantly correlated with poor overall survival in CRC patients.

**Conclusion:**

Our studies here provided evidence that RelB plays an oncogenic role and conveys chemo-resistance to 5-FU. RelB can be considered as an independent indicator of prognosis in CRC.

## Background

Colorectal cancer (CRC) is a leading cause of morbidity and mortality worldwide, and is a multistep genetic disorder. It ranks the third most common cancer among both men and women in the United States [1]. In China, CRC is the fifth leading cause of cancer deaths among both men and women [2]. Despite the achievements in the diagnosis and treatment of CRC acquired in recent years, the overall 5-year survival rate is still unfavored [3]. The development of CRC is a complex process, involving inactivation of several tumor suppressor genes and activation of proto-oncogenes. The main factors affecting the prognosis of CRC are metastasis, relapse, and chemo-resistance [4]. Therefore, a lot of efforts are desired to illuminate of the molecular mechanism underlying the CRC progression and metastasis, and to develop effective therapeutic target for CRC.

The nuclear transcription factor kappa B (NF-κB) has been identified as a nuclear factor binding to the kappa light chain enhancer in B cells in 1986 [5]. The NF-κB family includes RelA (p65), RelB, c-Rel, NF-κB1 (p50 and its precursor p105), and NF-κB2 (p52 and its precursor p100) [6]. NF-κB can be activated through the classical (canonical) and the alternative (or non-canonical) signaling pathways [7]. The classical NF-κB pathway involves activation of the IκB kinase (IKK) complex (composed of IKKα, IKKβ, and IKKγ subunits), leading to phosphorylation of IκB proteins. This pathway usually regulates the nuclear translocation activity of p50/RelA and p50/c-Rel heterodimers. In the alternative pathway, the NF-κB inducing kinase (NIK) activates IKKα, then leading to the phosphorylation and proteasome-mediated partial degradation of p100 to generate p52, resulting in the formation of RelB/p52 complexes. RelB/p52 heterodimers then translocate to the nucleus and activate target genes [8]. The role of the classical NF-κB activity has been extensively studied in a variety of human malignancies [9]. Many studies have focused on the function of the classical NF-κB pathway in CRC. In CRC, the NF-κB pathway plays a critical role in cancer related processes including cell proliferation, apoptosis, and metastasis [10]. The role of chronic inflammation in CRC is undisputed and the NF-κB pathway may serve as the link between inflammation and the tumorigenesis of colon epithelium [11]. Previous study shows that IKKβ-mediated NF-κB activity has a key role in the development of colitis-associated cancer using a mouse model of colitis-associated CRC [12]. Overexpression of the NIK- and IKK-β-binding protein (NIBP) can increase CRC metastases via classical NF-κB activity, which further upregulates matrix metallopeptidase 2 (MMP2) and matrix metallopeptidase 9 (MMP9) [13]. The classical NF-κB activity has also been implicated in the chemo-resistance and proteasome inhibition targeting NF-κB in CRC [14].

RelB is the main subunit of the alternative NF-κB signaling pathway, triggering effective transcription activation upon heterodimerizing with p52 [15]. Previous studies using the *RelB*^−/−^ mice have shown that RelB exerts crucial roles on numerous biological processes including lymphoid organogenesis, B cell maturation, T-cell homeostasis, and immune response. The lack of RelB cannot be functionally compensated by other NF-κB subunits [16–18]. Increasing studies have focused on the role of the alternative NF-κB activity, represented by RelB, in the tumourigenesis. The mRNA level of RelB is correlated with bladder cancer pathological, clinical stage, and lymph node metastasis [19]. The level of nuclear RelB expression correlates with prostate cancer patient’s Gleason score, suggesting that RelB is involved in prostate cancer progression [20]. RelB expression exhibits opposing effects of ascorbic acid in prostatic cancer and normal cells. RelB exerts a radio-protective role in aggressive prostate cancer cells through the induction of the manganese superoxide dismutase (*MnSOD*) gene [21]. Our previous studies have demonstrated that the RelB-silencing significantly attenuates the migration and invasion abilities of DU145 prostate cancer cells via the reduction of Integrin β-1 (ITGB1) [22]. Enhancer of zeste homology 2 (EZH2) can promote the transcriptional activation of RelB, driving self-renewal and tumor-initiating cell phenotype of triple-negative breast cancer cells [23]. Moreover, RelB is an independent prognostic factor for patients with non-small cell lung cancer (NSCLC). RelB promotes cell migration and invasion, and conveys radio-resistance to the NSCLC cells [24, 25]. Collectively, accumulated reports indicate that RelB functions importantly in the progression and chemo-sensitivity of various solid tumors.

The exact molecular mechanism of RelB in CRC remains unclear. The study here was aiming to define the significance of RelB in colon cancer cells. Our results indicated that RelB affected many cellular behaviors of DLD-1 colon cancer cells including proliferation, migration, invasion, and chemo-sensitivity. The expression of RelB was correlated with CRC clinical stage, tumor differentiation, and lymph node metastasis. RelB could be considered as an independent prognostic biomarker for CRC patients. Taken together, we provided evidences that RelB played an oncogenic role in CRC.

## Materials and methods

### Cell lines and culture condition

The human colon cancer cell lines DLD-1, HT-29, and Caco-2 were purchased from Shanghai Chinese Academy. All cells were cultured in RPMI-1640 media containing 10% fetal bovine serum (FBS, Gibco, USA), 100 U/ml penicillin, and 100 µg/ml streptomycin in a humidified atmosphere of 37 °C containing 5% CO_2_.

### Cell transfection

The short hairpin RNA (shRNA) specifically targeting the human *RelB* gene was designed and constructed by Invitrogen (Beijing, China). The sequences of RelB-shRNA are 275–293: *5′*-*GCACAGATGAATTGGAGAT*-*3′*. The shRNA-RelB was subcloned into the pSilencer3.1-H1-neo plasmid (Cat Nr. 5770, Thermo Scientific™, China), which was linearized by restriction endonucleases *Hin*dIII and *Bam*HI. DLD-1 colon cancer cells were pre-cultured to 60–80% confluence in 24-well plates and transfected using Lipofectamine 2000 (Cat Nr. 12566014, Thermo Scientific™, China) for 6 h. To obtain stably transfected clones, cells were selected in the medium containing G418 (800 ng/µl, Cat Nr. E859-5G, Amresco, USA).

### Western blot analysis and antibodies

Cells were collected and then lysed with a modified radioimmune precipitation assay (RIPA) buffer containing a protease inhibitor cocktail. Total protein and cytoplasmic/nuclear fractions were extracted, and denatured. Protein extracts were separated on 8–12% SDS-PAGE gels and further semi-electrically transferred into nitrocellulose membranes. After being blocked with 5% skim milk for 1 h at room temperature (RT), the membranes were incubated with appropriate primary antibodies (Abs) overnight at 4 °C. After washing with TBS-T buffer, the membranes were incubated with appropriate secondary Abs for 1 h at RT and scanned with an Odyssey^®^ infrared imaging system (LI-COR Biosciences, USA). Both primary and secondary Abs used in this study were diluted according to the manufacturer’s instructions. Band density was normalized to either β-actin or Lamin A/C expression. Abs against NF-κB p65 (C-20, sc-372), RelB (C-19, sc-226), c-Rel (N, sc-70), NF-κB p105/50 (H-119, sc-7178), NF-κB p100/52 (K-27, sc-298), IKBα (H-4), IKKα (B-8),and Lamin A/C (H-110, sc-20681) were purchased from Santa Cruz Biotechnology, lnc. Phospho-Akt Pathway Antibody Sampler Kit (#9916), Cell Cycle Regulation Sampler Kit (#9932) were purchased from Cell Signaling Technology, Inc. Abs against IKKβ (#2684), Phospho-IKKβ (#2694), Phospho-GSK-3β (#5558), Phospho-mTOR (#2971), Phospho-p70 S6 Kinase (#9205), MMP2 (D8N9Y), MMP9 (D603H), and Integrin β-1 (D2E5) were purchased from Cell Signaling Technology, Inc. β-actin Ab (AT0001) was purchased from Abgent (Suzhou, China). IRDye 680CW (#926-32222) and IRDye 800CW (#926-32210) secondary Abs were purchased from LI-COR Biosciences.

### RNA extraction and quantitative real-time PCR (qRT-PCR)

Total RNA was isolated using TRIzol reagent (Tiangen Biotech Co., Ltd., Beijing, China). RNA yield and purity were determined by Nanodrop-1000 spectrophotometer (Thermo Fisher Scientific, China). The absorbance ratio (A260/280) of all samples ranged from 1.8 to 2.0. Total RNA (2 µg) was reverse-transcribed into cDNA using Moloney Murine Leukemia Virus (M-MLV) (Cat Nr. 28025013, Promega, China) according to the manufacturer’s instructions. cDNA was then amplified by qRT-PCR assay though a SYBR Green PCR kit (Applied Biosystems, Shanghai, China) with a LightCycler 480 System (Roche, China). The mRNA expression level was calculated via Pfaffl method using *β*-*actin* as the internal control. Primers for qRT-PCR were designed using Primer-BLAST (Pubmed) and synthesized from Invitrogen.

### NF-κB DNA-binding capability assay

NF-κB DNA-binding capability was quantified using a Trans^AM^ NF-κB family transcription factor assay kit (Cat Nr. #43296, Active Motif, Carlsbad, CA, USA). Briefly, 5 µg of nuclear extracts were incubated in a 96-well plate coated with immobilized NF-κB consensus oligonucleotides (5′-GGGACTTTCC-3′) for 1 h at RT. Then captured complexes were incubated with individual NF-κB antibodies (1:1000) for 1 h, and subsequently with HRP-conjugated secondary antibody (1:1000) for 1 h. After colorimetric reaction, the absorbance was read as optical density (OD) value at 450 nm.

### Cell growth assay

The cell growth rates were detected by an x-Celligence RTCA instrument (Roche Diagnostics, China). In this assay, cells were seeded in an E-plate at a density of 5000 cells per well in 100 µl RPMI-1640 media containing 10% FBS. Impedance of cells for indicated times were continuously monitored by the system for 72 h and the value was measured as ‘cell index’. The data were analyzed by RTCA software 1.2. The x-Celligence system was also used to examine the effects of 5-Fluorouracil (5-FU, Cat Nr. F6627, Sigma Chemical) on cell growth. Cells were pro-cultured in an E-plate (5000 cells per well) in 100 µl RPMI-1640 media containing 10% FBS for 24 h. And cells were then treated with different concentrations of 5-FU (0–200 µM). Impedance of cells for indicated times were continuously monitored by the system for 48 h and the value was measured as ‘normalized cell index’. The dosage of 5-FU for 50% inhibition of proliferation (IC50) was analyzed by the RTCA software 1.2.

### CCK-8 assay

Cell proliferation was also measured using a Cell Counting Kit-8 (CCK-8, Dojindo, Kumomoto, Japan) assay. In the assay, cells were cultured in 96-well plates (3000 cells/well) and tested at the indicated times according to the manufacturer’s instructions. The absorbance of 450 nm was measured to calculate cell growth rates. Each experiment was repeated in triplicate.

### Brdu cell proliferation assay

Brdu cell proliferation assay kit (Cat Nr. 2750, Merck Millipore, Germany) was used to examine the cellular proliferation. In brief, cells were cultured in 96-well plates for 24 h and 10 µl Brdu was added for 5 h’ incubation. Then, the Brdu-labeled cells were fixed, and DNA was denatured. The cells were then incubated with peroxidase-conjugated anti-Brdu antibody for 1 h at RT. The immune complex was detected using a tetramethyl benzidine substrate reaction, and OD value at 450 nm was measured using spectrophotometer microplate reader (Biotek, USA). Each experiment was repeated in triplicate.

### Colony formation assay

For the colony formation assay, 1000 cells were seeded in 6-cm dishes, cultured in a humidified atmosphere of 37 °C containing 5% CO_2_ for 2 weeks, and then stained with Giemsa. Colonies containing more than 50 cells were counted, and the efficiency was calculated as a percentage of inoculated cells. Each experiment was repeated in triplicate.

### Cell apoptosis assay

Cells were cultured in 6-well plates for 0, 24, 48, and 72 h and were stained with AnnexinV together with propidium iodide (PI) using APC-AnnexinV Binding apoptosis assay kit (Cat Nr. 22837, AATBioquest, USA). Cells were incubated for 15 min at RT in the dark. Cell apoptosis was examined by a FACSCalibur™ cytometer (BD Biosciences, USA).

### Cell cycle assay

Cells were harvested and fixed with 70% ethanol overnight at 4 °C. Subsequently, the single cell suspensions were prepared to stain with PI (Cat Nr. P4170, Sigma, Germany) containing RNaseA (Cat Nr. 12091-021, Invitrogen, USA) according to the manufacturer’s instructions. Cell cycle (2 × 10^4^ cells) was measured by a FACSCalibur™ cytometer. The percentage of cells in the G_0_–G_1_, S, and G2-M phases was calculated by ModFit LT cell cycle analysis software.

### Cell migration and invasion assay using an x-Celligence system

Cells were seeded into the upper chamber in a CIM-plate assembled with the serum-free RPMI-1640 media. 200 μl RPMI-1640 media containing 10% FBS was added to each well of the lower chamber. For cell invasion assay, the Matrigel (Cat Nr. 356234, BD Biosciences, China) was diluted (1:40) using serum-from RPMI-1640 media. Wells of the upper chamber were pre-coated with Matrigel (30 µl) for 4 h. Cell migration or invasion through Matrigel towards the lower chamber was continuously monitored by an x-Celligence system, and data were collected and analyzed by RTCA software 1.2.

### Cell migration and invasion assay using a Transwell system

Cell migration and invasion assays were also performed using 24-well Transwell plates (Falcon cell culture inserts, 8-μm pore size, BD Biosciences, USA). To eliminate the influences of cell proliferation on cell migration and invasion, the cells were pre-treated with Mitomycin C (1M, CAS 50-07-7, Santa Cruz Biotechnology) for 1 h. For the migration assay, cells (5 × 10^4^) were seeded into the upper chamber of Transwell inserts containing serum-free RPMI-1640 media. 600 µl of RPMI-1640 media with 10% FBS was added as the chemotactic factor into the lower chamber. For the invasion assays, cells (10 × 10^4^) were seeded into the upper chamber of Transwell inserts pre-coated with 50 µl Matrigel. Upon incubation at 37 °C for 24 h, cells remaining on the upper surface were removed by swabs. Cells on the filter surface were fixed with 4% paraformal dehydrate for 20 min, and stained with 0.1% crystal violet for 15 min. Then the numbers of migratory or invasive cells were counted and photographed with a light System microscope IX71 (Olympus, Japan).

### Wound-healing assay

Artificial homogeneous wounds were created using a 200-μl micropipette tip and washed three times with PBS. Cells were then cultured with RPMI-1640 media supplemented with 10% FBS and photographed at 0, 24, 48, and 72 h with a light System microscope IX71 (Olympus, Japan).

### Clinical samples

The expression of RelB in CRC tissue was determined using tissue microarray [TMA, Lot Nr. XT16-027, Shanghai Outdo Biotech Company from the National Human Genetic Resources Sharing Service Platform (2005DKA21300)]. Use of patient samples and clinical data in this study was approved by the Ethics Committee of Shanghai Outdo Biotech Company. TMA consists of 87 paired CRC and para-carcinoma tissues. All patients underwent operation from November 2009 to May 2010. Patients’ follow-up information was obtained from 2009 to 2015. The final follow-up date was July 2015.

### Immunohistochemistry (IHC)

The sections of TMA were blocked by hydrogen peroxide and serum, and then incubated with primary Abs of RelB (1:200 dilutions). PBS was used as negative control for primary Abs. Staining and processing were performed with the GTVision™ III Detection System/Mo&Rb (Cat Nr. GK500705, Shanghai) according to the manufacturer’s instructions. IHC-staining results were analyzed by two experienced pathologists under a microscope BX51 (Olympus, Japan). The scores were as follows: 0 for no staining; 1+ for light; 2+ for moderate; 3+ for strong. The distribution of positive staining was scored as the percentages of labeled cells for five groups: 0: no staining; 1: < 25% staining; 2: 26–50% staining; 3: 51–75% staining; and 4: 75–100% staining. The product of the intensity and extent grades ≥ 4 of positive cells was considered as high-expression, and the score of 0–3 of positive cells was regarded as low-expression.

### Statistical analysis

All analyses were analyzed by SPSS 24.0 and GraphPad Primer5 software. The measured data of normal distribution were expressed as mean ± SD, and differences between groups were examined by Student’s *t*-test. A Pearson’s Chi square or Fisher’s exact test was used to analyze the correlation between RelB expression and clinic-pathological characteristics. Kaplan–Meier test were used for survival analysis. Multivariate Cox proportional risk models were performed to value the effect of RelB expression levels on disease-specific survival. A value of *p *< 0.05 was considered statistical significant. * for *p *< 0.05, ** for *p *< 0.01, *** for *p *< 0.001.

## Results

### Introduction of RelB-shRNA into DLD-1 colon cancer cells

**Fig. 1:**
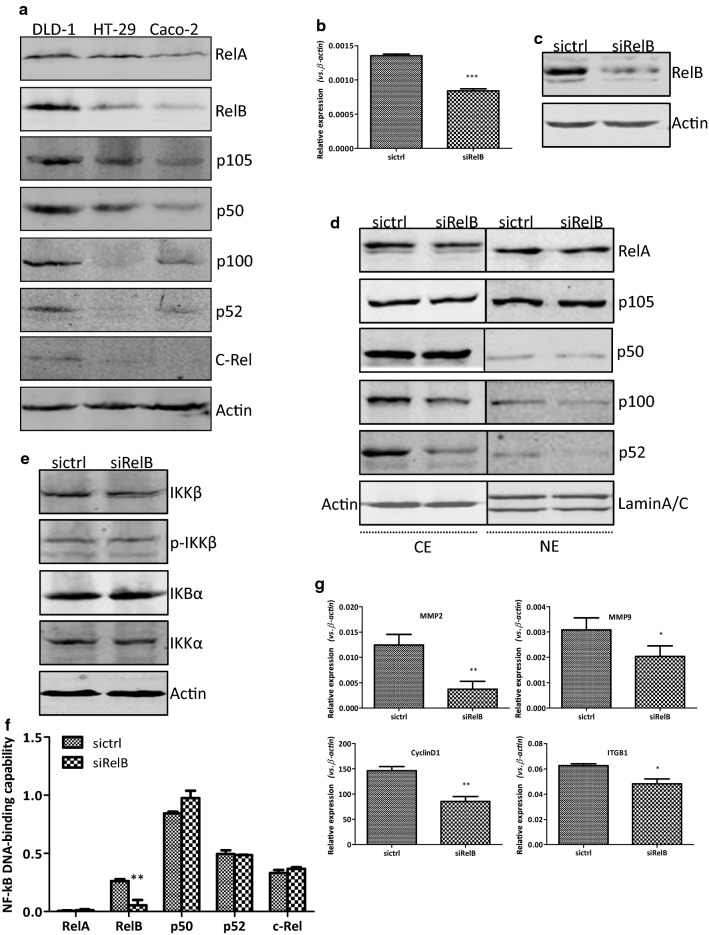
Establishment of RelB-silencing DLD1 cells. **a** Western blotting analysis of the protein expression of NF-κB subunits in DLD-1, HT-29, and Caco-2 cells. The level of each protein was normalized against Actin. **b** Relative RelB mRNA expression levels were detected using qRT-PCR. β-Actin normalized gene expression, measured in triplicates was displayed. **c** Western blotting analysis for protein levels of RelB expression. Protein expression was normalized against Actin. **d** RelB-silencing affects the expression of other individual NF-κB subunits. Western blotting analysis of the protein expression in cytoplasmic and nuclear extracts was normalized against Actin and Lamin A/C, respectively. **e** Western blotting analysis of the protein expression of IKKβ, p-IKKβ, IKBα, and IKKα in siRelB and sictrl cells. Protein levels were normalized against Actin. **f** The DNA-binding activity of nuclear extracts was detected and quantified using a Trans^AM^ NF-κB family transcription factor assay kit. **g** The mRNA expression levels of specific target genes downstream of NF-κB pathway were detected using qRT-PCR. β-Actin normalized gene expression, measured in triplicates was displayed. Data are shown as mean ± SD form three individual experiments. *p < 0.05; **p < 0.01; ***p < 0.001

The endogenous expression of all NF-κB subunits in the whole-cell extracts was examined by Western blotting analysis in the three colon cancer cell lines including DLD-1, HT-29, and Caco-2. As shown in Fig. 1a, the expression levels of each NF-κB subunits were quite different among the three colon cancer cell lines. All the NF-κB subunits could be detected in the DLD-1 cells albeit at different levels. The expression of RelA, RelB, p105, and p50 could be detected in the HT-29 cells, while the expression of c-Rel, p100, and p52 not detected. All the NF-κB subunits except of c-Rel could be detected in the Caco-2 cells.

To investigate the function of RelB on the biological cellular behaviors of colon cancer cells, the DLD-1 cells with high RelB expression were preferred for the following experiments. The DLD-1 cells were transfected with plasmid carrying either RelB-shRNA or control-shRNA respectively. Cells were cultured in the presence of G418 until monoclones formed. The expression of RelB in the DLD-1 monoclones was examined by both qRT-PCR and Western blotting analysis and the representative results were present here. As shown in Fig. 1b, the mRNA expression of *RelB* was decreased about twofold in the DLD-1-siRelB cells (transfected with the RelB-shRNA plasmid) compared with that in the DLD-1-sictrl cells (transfected with the control-shRNA plasmid). Similarly, the RelB expression at protein level was much lower in the DLD-1-siRelB cell compared with that in the DLD-1-sictrl cells (Fig. 1c).

To examine whether the RelB-silencing affected the NF-κB signaling, the Western blotting analysis was performed. As shown in Fig. 1d, the expression of RelA, p50, p105, and c-Rel in both cytoplasmic extracts (CE) and nuclear extracts (NE) stayed unchanged, while the expression of p100 and p52 was also reduced in CE and NE in the DLD-1 cells lacking the expression of RelB. The expression of certain upstream molecules of the NF-κB signaling, such as IKKβ and phosphorate-IKKβ, was not influenced by the RelB-silencing in the DLD-1 cells (Fig. 1e). To further investigate the effects of the RelB-silencing on NF-κB DNA-binding capability, an ELISA-based NF-κB activity assay was performed. Compared to that in the DLD-1-sictrl cells, the average RelB DNA-binding capability in NE of the DLD-1-siRelB cells was considerably reduced (*p *< 0.01). Meanwhile, the average p52 DNA-binding capability was slightly reduced, albeit with no statistical significance. The average DNA-binding capabilities of RelA, p50, and c-Rel in the DLD-1-siRelB and DLD-1-sictrl cells were comparable (Fig. 1f). In addition, the mRNA levels of important genes regulated by the alternative NF-κB signaling were examined by qRT-PCR. As shown in Fig. 1g, the mRNA expression of the *MMP2* (3.4-fold, *p *< 0.01), *MMP9* (1.5-fold, *p *< 0.05), *CyclinD1* (1.7-fold, *p *< 0.01), and *ITGB1* (1.3-fold, *p *< 0.05) genes was decreased at different levels in the DLD-1-siRelB cells compared with that in the DLD-1-sictrl cells. Collectively, these results indicated that a successful RNA-interference of the *RelB* gene was created in the DLD-1 cells.

### RelB affects the DLD-1 cell growth

**Fig. 2:**
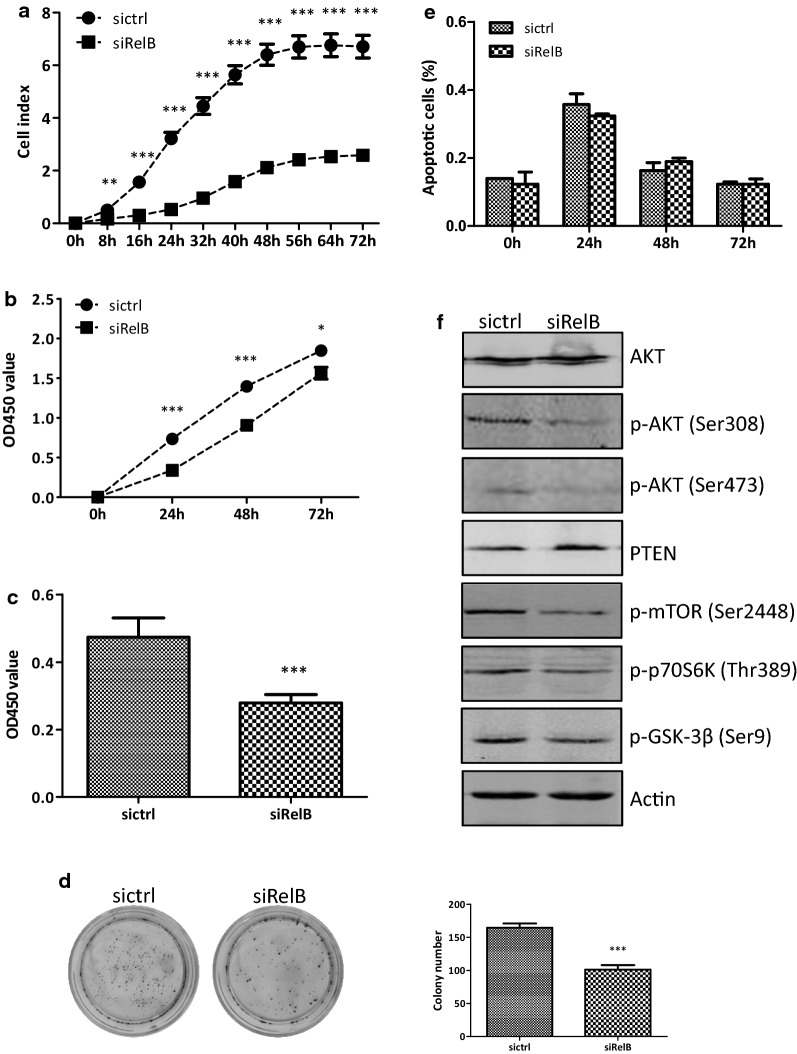
RelB-silencing inhibits the DLD-1 cell growth. **a** The cell growth rates were monitored by a real-time x-Celligence system for 72 h. **b** Cell proliferation was detected by CCK-8 assay. OD450 was measured using spectrophotometer microplate reader at 24, 48, and 72 h. **c** Cell proliferation was detected by Brdu assay. OD450 was measured after transfection with siRNA for 24 h. **d** Cell proliferation was evaluated by colony formation assay. 1000 cells were seeded in 6-cm dishes and cultured for 2 weeks. Colonies containing > 50 cells were counted. **e** Cell apoptosis assay was examined by Flow cytometry at 0, 24, 48, and 72 h. **f** Western blotting analysis of the protein expression of total AKT, p-AKT^Ser473^, p-AKT^Ser308^, p-mTOR^Ser2448^, p-p70S6K^Thr389^, PTEN, and p-GSK-3β^Ser9^ in siRelB and sictrl cells. Protein levels were normalized against Actin. *p < 0.05; **p < 0.01; ***p < 0.001

In order to explore the effects of the RelB-silencing on cell growth, the cellular growth was continuously monitored by a real-time x-Celligence system using E-plates. As shown in Fig. 2a, the cell growth curves of the DLD-1-siRelB and DLD-1-sictrl cells were clearly separated during the 72 h’s continuous monitoring. The DLD-1-siRelB cells grew much slower than the DLD-1-sictrl cells. There were statistically significant differences between the two established cell lines after culturing for 8 h and later (*p *< 0.01 at 8 h time point, *p *< 0.001 from 16 h). Cell proliferation was examined by a CCK-8 assay (Fig. 2b). The OD450 values of the DLD-1-sictrl cells were 0.73 ± 0.01, 1.40 ± 0.05, and 1.85 ± 0.07, while the values of the DLD-1-siRelB cells were 0.34 ± 0.00, 0.90 ± 0.04, and 1.56 ± 0.08 at 24, 48 and 72 h, respectively. The OD450 values were clearly decreased in the DLD-1-siRelB cells, and there were statistically significant differences between the two cell lines at 24 h (*p *< 0.001), 48 h (*p *< 0.001), and 72 h (*p *< 0.05). A Brdu assay was carried out to examine the cellular proliferation as well. The OD450 value of the DLD-1-siRelB cells (0.28 ± 0.01) was lower than that of the DLD-1-sictrl cells (0.47 ± 0.03) at 24 h (*p *< 0.01, Fig. 2c). The results got from the Brdu assay were in line with the CCK-8 assay data. In colony formation assay, colonies containing at least 50 cells from the DLD-1-sictrl cells were 165 ± 4, while 101 ± 4 colonies from the DLD-1-siRelB cells (Fig. 2d). The colony formation efficiency of the DLD-1-siRelB cells (10.1%) was fewer than that of the DLD-1-sictrl cells (16.5%, *p *< 0.01). Taken together, these data clearly demonstrated that the RelB-silencing in the DLD-1 cells affected cell proliferation.

RelB has been reported to function in the spontaneous or radiotherapy-induced apoptosis in tumor cells.. As shown in Fig. 2e, the percentages of spontaneous apoptosis were quite low, 0.32 ± 0.00%, 0.19 ± 0.00%, and 0.12 ± 0.00% in the DLD-1-siRelB group, and 0.36 ± 0.02%, 0.16 ± 0.01%, and 0.12 ± 0.00% in the DLD-1-sictrl group at 24, 48 and 72 h, respectively. There were no significant differences between the two established cell lines at all time points. Therefore, RelB unlikely affected spontaneous apoptosis; rather, RelB promoted the cellular proliferation of the DLD-1 cells, which further quickened the cell growth.

Protein kinase B (AKT, as known as PKB)/mammalian target of rapamycin (mTOR) signaling pathway regulates various biological processes including cell proliferation, survival, and angiogenesis. To verify whether the signaling was influenced in the DLD-1 cells lacking RelB expression, Western blotting assay was performed to examine the important molecules in the signaling. As shown in Fig. 2f, the total AKT stayed unchanged. The expression of phosphor-AKT (Thr308) and phosphor-AKT (Ser473) in the DLD-1-siRelB cells were evidently reduced compared to that of the DLD-1-sictrl cells. Phosphate and tension homology deleted on chromosome ten (PTEN), a negative regulator of the AKT signaling, was induced in the DLD-1-siRelB cells. mTOR is a key kinase downstream of AKT. The phosphorylation of mTOR at Ser2448, regulated directly by AKT and leading to the activation of mTOR, was deduced in the DLD-1-siRelB cells. Ribosomal protein S6 kinase beta-1 (S6K1, known as p70S6 K), a downstream target of mTOR signaling, was also deduced in the DLD-1-siRelB cells. Glycogen syntheses kinase 3β (GSK-3β) could be inactivated by phosphorylation of serine at residue 9, triggered by the AKT and mitogen-activated protein kinase (MAPK) signaling pathways. The diminished phosphorylation of GSK-3β at Ser9, leading to GSK-3β activation, was observed in the DLD-1-siRelB cells. Take together, the data here showed that the AKT/mTOR signal pathway was inactivated by the RelB-silencing in the DLD-1 cells, which contributed to the impaired cell proliferation.

#### RelB affects 5-FU response

**Fig. 3:**
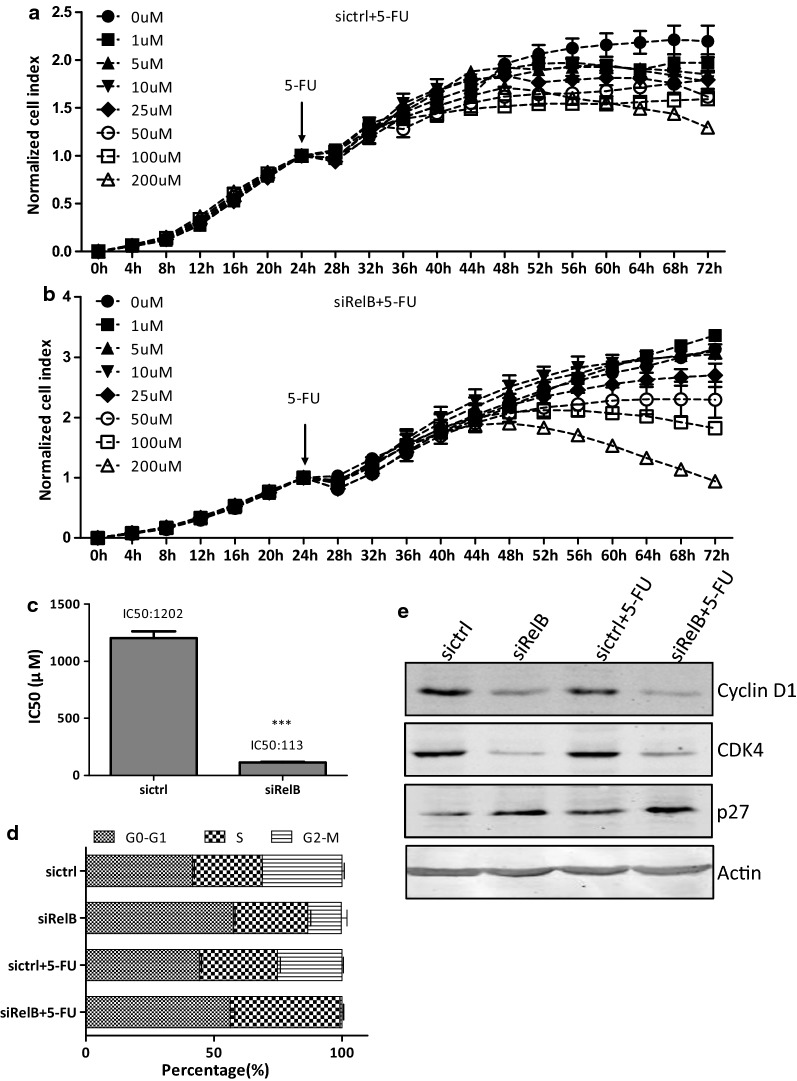
RelB-silencing enhances cytotoxic effect of 5-FU toward DLD-1 cells. **a**, **b** X-Celligence system was used to examine the effects of 5-FU on cell growth. Cells were pro-cultured in an E-plate (5000 cells per well) for 24 h and then different concentrations of 5-FU (0, 1, 5, 10, 50, 100, 200 µM) were added. Impedance of cells for indicated times were continuously monitored by the system for 72 h and the value was measured as ‘normalized cell index’. **c** The dosage of 5-FU for IC50 was analyzed at the time of treated with 5-FU for 48 h. **d** Cell cycle assay determined by flow cytometry. The distribution of the G_0_–G_1_, S, and G2-M phase were displayed in the table. **e** Western blotting analysis of the expression of cell cycle related-proteins. Protein levels were normalized against Actin.*p < 0.05; **p < 0.01; ***p < 0.001

5-FU is a powerful chemo-therapeutic agent used wildly in treating diverse solid tumors, including CRC [26]. To examine whether the RelB-silencing affected the 5-FU efficacy on the DLD-1 colon cancer cells, the cell growth was monitored by the x-Celligence system. Both the DLD-1-sictrl and DLD-1-siRelB cells were treated with different concentrations of 5-FU (0, 1, 5, 10, 25, 50, 100, and 200 μM) for 48 h. The cell growth rate of the DLD-1-sictrl and DLD-1-siRelB cells upon treated with 5-FU was decreased in a dose-dependent manner (Fig. 3a, b). The IC50 of 5-FU for the DLD-1-siRelB cells was 113 μM (95% CI 95–138 μM), for the DLD-1-sictrl cells was 1202 μM (95% CI 403–11258 μM). The IC50 of 5-FU for the DLD-1-siRelB cells was significantly lower compared with that of the control cells, and there was statistically significant difference (*p *< 0.001, Fig. 3c). The results suggested that of the RelB-silencing enhanced the sensitivity to 5-FU of the DLD-1 colon cancer cells.

Cell cycle distribution was examined by PI-staining which was followed by flow cytometry analyses. The distributions of G_0_–G_1_, S, and G_2_-M phase in the DLD-1-sictrl cells were 41.69 ± 0.41%, 27.65 ± 0.06%, and 31.15 ± 0.47%, while those in the DLD-1-siRelB cells were 57.65 ± 0.36%, 28.89 ± 0.71%, and 13.16 ± 1.30%, respectively (Fig. 3d). Cell cycle progression of the DLD-1-siRelB cells was notably arrested in the G_0_–G_1_ phase (*p *< 0.001). Cyclin D1 can form a complex with and function as a regulatory subunit of cyclin-dependent kinase 4 (CDK4) or cyclin-dependent kinase 6 (CDK6), whose activity is required for cell cycle progression through the G_1_ phase. Cyclin-dependent kinase inhibitor 1B (p27Kip1) binds to and prevents the activation of Cyclin D1-CDK4 complexes, and thus controls the cell cycle progression at G_1_ phase. As shown in Fig. 3e, both Cyclin D1 and CDK4 were clearly decreased while p27^Kip1^ was increased in the DLD-1-siRelB cells. RelB likely affected several cell-cycle regulatory molecules, especially Cyclin D1, CDK4, and p27^Kip1^, which contributed together to the G_0_–G_1_ arrest.

Expose to 5-FU (113 μM) for 48 h, the distributions of G_0_–G_1_, S, and G_2_-M phase in the DLD-1-sictrl cells were 44.43 ± 0.44%, 30.31 ± 0.70%, and 25.26 ± 0.30%, while those in the DLD-1-siRelB cells were 56.35 ± 1.04%, 42.94 ± 0.70%, and 0.71 ± 0.40% (Fig. 3d). Importantly, treated with 5-FU caused cell accumulation in the S phase in both cell lines, from 27.65% to 30.31% (*p *< 0.05) in the DLD-1-sictrl cells, from 28.89% to 42.94% (*p *< 0.001) in the DLD-1-siRelB cells. The induction of the cell arrest in the S-phase was significant in the DLD-1-siRelB cells upon treated with 5-FU (14.05% vs. 2.66%, *p *< 0.001). In both DLD-1-sictrl and DLD-1-siRelB cells with or without 5-FU treatment, the expression of CDK4 and p27^Kip1^ stayed unchanged. However, the expression of Cyclin D1 was slightly decreased (Fig. 3e).

### RelB promotes cell migration and invasion

**Fig. 4:**
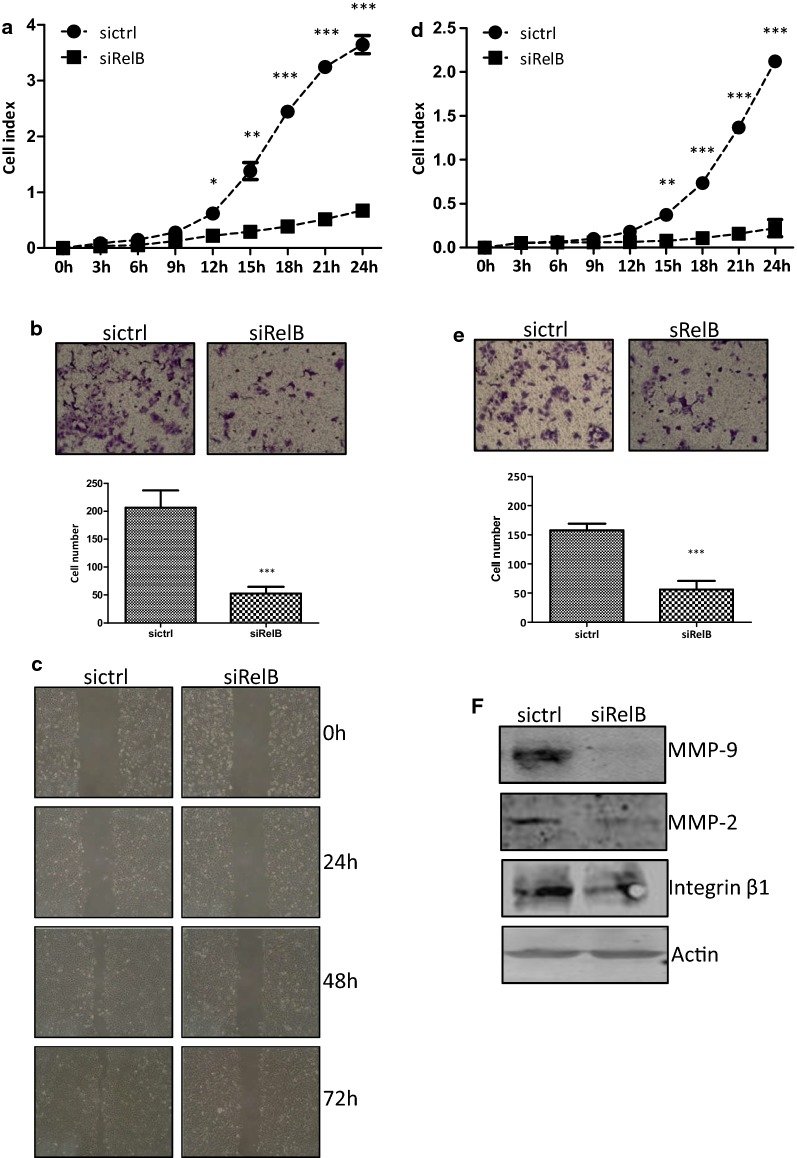
RelB-silencing hampers the migration and invasion abilities of DLD-1 cells. **a** The migration abilities of the two established cells were assessed by a real-timex-Celligence system for 24 h. **b** Representative images and data of a Transwell migration assay. The number of migratory cells were counted and photographed at 24 h. **c** Cell migration ability assessed by wound healing assay at 0, 24, 48, and 72 h. **d** The invasion abilities of the two established cells were detected by a real-time x-Celligence system using CIM-plate pre-coated with Matrigel (1:40) and Cell invasion was continuous monitored for 24 h. **e** Representative images and data of a Transwell invasion assay. Transwell chambers were pre-coated with Matrigel (1:8) and the number of invasive cells were counted and photographed at 24 h. **f** Western blotting analysis of the protein expression of MMP9, MMP2, and ITGB1 in siRelB and sictrl cells. Protein levels were normalized against Actin.*p < 0.05; **p < 0.01; ***p < 0.001

To investigate whether RelB played a role in cell migration in the DLD-1 cells, cells were continuously monitored by the x-Celligence system using CIM-plate for 24 h. As shown in Fig. 4a, the DLD-1-siRelB cells migrated dramatically slower than the DLD-1-sictrl cells. There were statistically significant differences between the two established cell lines from 12 to 24 h (*p *< 0.05 at 12 h, *p *< 0.01 at 15 h, *p *< 0.001 at 18, 21, and 24 h). Transwell inserts were also used to examine the role of RelB on the migration abilities of DLD-1 cells. Cells which had migrated the inserts were counted and photographed after 24 h. The number of migrated DLD-1-siRelB cells was 52 ± 7, less than that of the migrated DLD-1-sictrl cells, 206 ± 18 (*p *< 0.001, Fig. 4b). Wound healing assay was also carried out, and photographs were taken under microscope at 0, 24, 48, and 72 h, respectively. The DLD-1-siRelB cells migrated from the edge of the scratch toward the scratch centre much slower than the DLD-1-sictrl cells (Fig. 4c). Taken together, these results suggested that the RelB-silencing hampered the migratory ability of DLD-1 cells.

The invasion ability was detected also used the real-time x-Celligence system with CIM-plate pre-coated with Matrigel. As shown in Fig. 4d, the DLD-1-siRelB cells invaded the Matrigel much slower than the DLD-1-sictrl cells. There were statistically differences between the two established cell lines from 15 to 24 h (*p *< 0.01 at 15 h, *p *< 0.001 at 18, 21, and 24 h). Transwell inserts, pre-coated with Matrigel, were also used to investigate whether RelB affected the invasive ability of DLD-1 cells. The number of invaded DLD-1-siRelB cells was 56 ± 9, significantly fewer than that of the invaded DLD-1-sictrl cells, 157 ± 6 (*p *< 0.001, Fig. 4e). The RelB-silencing markedly impaired the cell invasion capacity of DLD-1 cells.

A variety of molecules are involved in regulating cellular migration and invasion of cancer cells, including the MMPs. As shown in Fig. 4f, the protein expression of MMP2 and MMP9 in the DLD-1-siRelB was clearly decreased compared with that of the DLD-1-sictrl cells. The mRNA levels of the *MMP2* and *MMP9* genes were reduced as well (Fig. 1g). Meanwhile, the decreased Integrin β1 expression was observed in the DLD-1-siRelB cells. These results suggested that RelB promoted the DLD-1 cell migration and invasion abilities, likely due to regulating the expression of MMP2, MMP9, and Integrin β-1.

### Relationship between RelB and clinic-pathological features of CRC patients

**Fig. 5:**
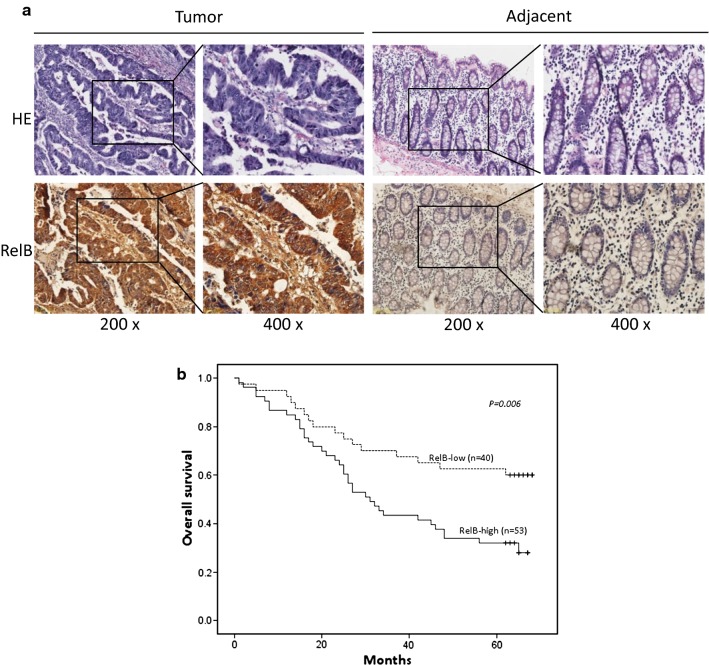
Clinical significance of RelB expression in CRC tissues. **a** Representative images of RelB expression in CRC tissues and adjacent colorectal mucosas were shown by IHC (×200 and ×400). **b** Kaplan–Meier curves that depict the 5-year overall survival in CRC patients. Patients were divided into two groups according to the RelB expression with significantly different prognosis (n = 93, *p *= 0.006). RelB-low represented the RelB low expression group; RelB-high represented the RelB high expression group

To assess the putative clinical significance of RelB expression in CRC patients, RelB expression was identified in CRC tissues and adjacent colorectal mucosa of 93 patients by IHC staining. Representative images of RelB expression in CRC and adjacent non-neoplastic tissues were present in Fig. 5a. RelB could be detected in both the nucleus and cytoplasm fractions of CRC cells. The expression of RelB was highly expressed in the CRC tissue while the expression of RelB was barely detected in the adjacent colorectal mucosa. According to the RelB expression, 93 patients were divided into two groups, RelB-high (53/93, 56.9%) and RelB-low (40/93, 43.1%).

As shown in Table 1, the clinico-pathological association study demonstrated that the expression of RelB was positively correlated with depth of tumor invasion (*p *= 0.018), lymph node metastasis (*p *< 0.001), metastasis stage (*p *= 0.031), and pTNM stage (*p *= 0.001). The expression of RelB was not correlated with age, gender, site of origin, or differentiation status of CRC patients. Kaplan–Meier analyses indicated that CRC patients with high-RelB expression were significantly correlated with a poorer overall survival (OS) than those with low-RelB expression. (*p *= 0.006, Fig. 5b).

**Table 1:** Relationship between RelB expression and clinico-pathological characteristics

Characteristics	Total N = 93	RelB expression		*p* value
		High N = 53	Low N = 40	
Age (years)				
≤ 60	34	18	16	0.549
> 60	59	35	24	
Gender				
Male	52	28	24	0.491
Female	41	25	16	
Site				
Right	34	19	15	0.915
Left	53	29	24	
Unknown	6	5	1	
Differentiation				
Well	2	1	1	0.332
Moderate	73	39	34	
Poor	18	13	5	
Depth of tumor invasion				
T1	0	0	0	0.018*
T2	5	1	4	
T3	64	32	32	
T4	16	13	3	
Unknown	8	1	7	
Lymph nodes				
N0	51	21	30	< 0.001***
N1	28	22	6	
N2	9	9	0	
Unknown	5	1	4	
Metastasis				
Yes	4	4	0	0.031*
No	89	49	40	
pTNM stage				
I	4	1	3	0.001***
II	41	14	27	
III	35	29	6	
IV	4	4	0	
Unknown	9	5	4	

A value of *p* < 0.05 was considered statistical significant. * *p* < 0.05, ** *p* < 0.01, *** *p* < 0.001

Univariate survival analyses indicated that tumor differentiation (*p *< 0.001), N stage (*p *= 0.001), M stage (*p *= 0.001), pTNM stage (*p *< 0.001), and RelB expression (*p *= 0.002) had statistical significances (Table 2). Multivariate Cox regression analyses indicated that tumor differentiation and RelB expression had statistical significances, with hazard ratio (HR) of 2.115 for tumor differentiation (95% CI 1.044–4.286, *p *= 0.038) and 2.996 for RelB (95% CI 1.848–6.047, *p *= 0.002). These analyses indicated that poor tumor differentiation and high RelB expression were independent factor for shorter OS in CRC patients.

**Table 2:** Univariate and multivariate analyses of OS in patients with CRC

Characteristics	Univariate analysis		Multivariate analysis	
	HR (95%) CI	*p* value	HR (95%) CI	*p* value
Age (≥ 60 vs. < 60)	1.525 (0.838–2.776)	0.167		
Gender (male vs. female)	0.835 (0.487–1.432)	0.512		
Site (right vs. left)	0.738 (0.415–1.312)	0.738		
Differentiation (poor vs. well, moderate)	3.034 (1.664–5.533)	< 0.001***	2.115 (1.044–4.286)	0.038*
T stage (T4 vs. T2, T3)	1.574 (0.804–3.079)	0.186		
N stage (N1, N2 vs. N0)	2.725 (1.550–4.793)	0.001***		
M stage (M1 vs. M0)	2.725 (1.550–4.793)	0.001***		
pTNM stage (III, IV vs. I, II)	2.916 (1.635–5.201)	< 0.001***		
RelB expression (high vs. low)	2.657 (1.438–4.907)	0.002**	2.996 (1.484–6.047)	0.002**

A value of *p* < 0.05 was considered statistical significant. * *p* < 0.05, ** *p* < 0.01, *** *p* < 0.001

## Discussion

The NF-κB signaling pathway is involved in multiple steps of carcinogenesis, such as initiation, proliferation, survival, metastasis, and chemo-resistance [6]. Chronic inflammation is considered as an important carcinogenic mechanism leading to the occurrence of CRC and the NF-κB family members have been served as the bridge between inflammation and the tumourigenesis of colon epithelium [11, 27]. Studies have demonstrated that activation of targets of the NF-κB signaling pathway promote CRC metastasis and connect the inflammatory processes to carcinogenesis [10]. Many efforts have been made to develop NF-κB inhibitors, such as drugs targeting IKKs [28]. However, these drugs have many off-target effects on other signaling pathways. Thus, targeting specific NF-κB signaling component may help to overcome these obstacles [8]. RelB is involved in the initiation, progression, and chemo-resistance of several solid tumors including prostate, breast, endometrium, bladder, laryngeal, and non-small cell lung cancer [29–32].

The functions of RelB in DLD-1 colon cancer have not been addressed. The RelB-silencing slowed down the DLD-1 cell growth. The retarded cell growth in vitro in the absence of RelB expression was largely due to the reduced cell proliferation, which was attributed to the decreased phosphor-AKT and phosphor-mTOR. These observations pinpointed that RelB affected DLD-1 cell proliferation by regulating the AKT/mTOR signaling pathway. AKT, a serine/threonine-specific protein kinase, regulates cell proliferation and survival via phosphorylating and activating or inactivating downstream molecules. The results here are similar to previous finding that emphasizes the role of the alternative NF-κB pathway in cell proliferation, regulated by the AKT/mTOR signaling pathway [33]. In SPC-A1 lung cancer cells, cell proliferation is suppressed by the RelB-silencing. The volume and weight of subcutaneous tumors established by the subcutaneous xenograft model using the RelB-silencing SPC-A1 cells are also reduced [25]. Increased RelB expression enhances endometrioid adenocarcinoma (EEC) cell growth by regulating cell proliferation, leading to endometrial cell tumourigenicity [32]. The regulation between RelB and the AKT signaling requires further investigations. STI571, a tyrosine kinase inhibitor, enhances RelB nuclear translocation in LnCaP prostatic cancer cells. STI571 can inhibit the PI3K-AKT-IKKα pathway in PC-3 prostatic cancer cells by decreasing the phosphorylation of AKT at Ser473 [34].

RelB is reported as a crucial positive regulator of cell survival in multiple tumors, such as multiple myeloma, chronic lymphocytic leukemia, and prostatic cancer [22, 35–37]. However, different from previous reports, the RelB-silencing did not affect the survival of DLD-1 colon cancer cells. The constantly present RelA activity in the DLD-1 cells, in the presence of RelB-silencing, is certainly a potent survival regulator.

The cell cycle was significantly arrested in the G_0_–G_1_ phase in the DLD-1 cells lacking RelB expression, which was caused by decreased expression levels of Cyclin D1 and CDK4 together with up-regulated expression of p27^KIP1^. Cyclin D1 is expressed relatively early in the cell cycle and is essential for DNA synthesis. Studies demonstrate that NF-κB induces cell proliferation by regulating key cell-cycle regulatory genes including Cyclin D1 and CDKs [38]. It has been also shown that the RelB-silencing results in the arrest of EEC cells at the G_1_ phase via inhibiting Cyclin D1 [32]. Therefore, our results are consistent with the previous studies that the RelB-silencing caused G_0_–G_1_ arrest in the DLD-1 cells and inhibited cell proliferation. GS-3β mediated phosphorylation of Cyclin D1 plays a central role in the G_1_-to-S-phase cell-cycle transition [39]. The phosphorylation of GSK-3β, triggered by the AKT signaling pathway and led to GSK-3β inactivation, was clearly decreased in the DLD-1-siRelB cells.

5-Fluorouracil is one of the most commonly used chemotherapy drug for CRC. However, drug resistance is always acquired in CRC cells after 5-FU treatment [40]. Pharmacological targeting of NF-κB, such as inhibitor of the IKKβ to block NF-κB activation potentiate the cytotoxic effect of 5-FU  [41]. Recent studies show that inhibiting NF-κB signaling may be an effective strategy to reverse 5-FU resistance in CRC [42]. Whether RelB regulates the chemo-sensitivity of CRC cells to 5-FU has not been reported. In this study, the RelB-silencing enhanced cytotoxic effects of 5-FU on the DLD-1 cells. To further explore the possible mechanism, the cell cycle of the DLD-1 cells treated with 5-FU was examined. The RelB-silencing induced cell cycle accumulation in G_0_–G_1_ phase, and caused S-phase arrest after treatment with 5-FU. The RelB-silencing enhanced cytotoxic effects to 5-FU, by increasing the cell accumulation in S-phase. Many studies show that RelB plays an important role in terms of sensitivity to chemotherapy [43]. Treated with 5-FU induces S-phase arrest, which is related with upregulation of p27 and downregulation of Cyclin D1 [44]. Cyclin D1 is needed to allow the cells progress from G_1_ to S phase. With high level of Cyclin D1, cells can prevent 5-FU to be incorporated into the DNA [45]. Overexpression of Cyclin D1 protects CRC cells against 5-FU treatment [46]. Our results showed that there were more cells arrested in the S-phase in the absence of RelB after treated with 5-FU, likely due to reduction of Cyclin D1. Thus, the results suggested that the RelB-silencing enhanced 5-FU response by decreasing Cyclin D1 expression.

Metastasis is a main feature of advanced malignancies, and is an important factor in affecting the patient’s prognosis. A variety of molecular regulators are implicated to regulate the migration and invasion of cancer cells. Integrin β1, belonging to the family of heterodimeric transmembrane cell surface receptors, has multiple functions in cell adhesion and migration [47]. A recent study shows that miR-30e-5p overexpression inhibits CRC cell adhesion, migration, and invasion by decreasing Integrin β1 [48]. Loss of Integrin β1 expression in CRC shows poor survival and correlates with advanced clinical stage and lymph node metastasis [49, 50]. Previous studies show that the RelB-silencing suppresses migration and invasion abilities of DU145 prostatic cancer cells and SPC-A1 lung cancer cells by decreasing the expression of Integrin β1 [22, 25]. Our results are similar to previous findings, indicating that RelB regulates cell migration and invasion abilities in the DLD-1 cells. MMP2 and MMP9 are the representative members of MMPs. Tumor cells can alter extracellular matrix by overexpression of MMPs to promote invasion. Studies show that high expression levels of MMP2 in CRC tissues are correlated with reduced survival. The mRNA expression levels of MMP2 and MMP9 in CRC tissues are higher than that of normal mucosa [51, 52]. Recent studies shows tumor necrosis factor-like weak inducer of apoptosis (TWEAK) increases MMP9 expression to promote glioma cell invasion by activating the non-canonical NF-κB signaling pathway. RelB can promote invasion in glioma cells without affecting the activity of RelA and the classical NF-κB signaling [53]. Bioinformatics analysis also suggests that MMPs family genes are positively correlated with RelB in glioma tumorigenesis [54]. Moreover, Curcumin can inhibit colon cancer cell invasion by suppressing NF-κB-mediated transcriptional activation MMP9 [55]. NIK- and IKKβ-binding protein (NIBP) increases the CRC metastatic potential by activating the NF-κB pathway and increasing MMP2 and MMP9 expression [13]. Our results showed that the RelB-silencing suppressed the migration and invasion abilities of DLD-1 cells. The expression of MMP2 and MMP9, both at protein and mRNA levels, was decreased in the DLD-1-siRelB cells. The results here are consistent with previous studies, suggesting that RelB plays a role in the cell migration and invasion in DLD-1 cells by regulating the MMP2 and MMP9 expression.

In present study, we found that RelB expression was positively related to depth of tumor invasion, lymph node metastasis, metastasis stage, and pTNM stage. Therefore, the results indicated that RelB played a significant role in the metastasis of CRC, concomitant with in vitro experimental results. Furthermore, multivariate Cox regression analyses revealed that high expression of RelB was a poor prognostic marker in CRC, indicating that RelB can be considered as an independent prognostic factor. The TNM staging system is currently used to assess the prognosis of patients with CRC. For rectal cancer patients who undergo neo-adjuvant chemo-radiation, it is sometime unable to detect enough lymph nodes. Then TNM staging system is not always accurate. New biological markers of prognosis such as RelB may supplement its disadvantages.

## Conclusions

Our findings add an understanding to the unexplored functions of NF-κB subunit, RelB, in CRC. RelB significantly affects cell proliferation, cell migration and invasion, and chemo-sensitivity to 5-FU treatment via cell cycle alteration in DLD-1 cells. Moreover, RelB may function as a potential prognostic indicator of CRC patients. Therefore, RelB may represent a new strategy to CRC therapy. Our present study has several limitations. We used only one colon cancer cell line and did not perform any in vivo expression experiments, which will be performed in our future studies.

## Abbreviations

CRC: colorectal cancer
NF-κB: nuclear transcription factor kappa B
NIK: NF-κB inducing kinase
NIBP: NIK- and IKK-β binding protein
EZH2: enhancer of zeste homology 2
5-FU: 5-fluorouracil
NSCLC: non-small cell lung cancer
FBS: fetal bovine serum
shRNA: short hairpin RNA
Abs: antibodies
qRT-PCR: quantitative real-time PCR
CCK-8: Cell Counting Kit-8
PI: propidium iodide
PBS: phosphate-buffered saline
TMA: tissue microarray
IHC: immunohistochemistry
SD: standard deviation
OS: overall survival
CE: cytoplasmic extract
NE: nuclear extract
OD450: optical density at 450 nm
IC50: 50% inhibition of proliferation
MMPs: matrix metalloproteinase family
HR: hazard ratio
TWEAK: tumor necrosis factor-like weak inducer of apoptosis
